# Type-IV intestinal atresia: A challenging congenital anomaly with successful management

**DOI:** 10.12669/pjms.42.(ICON26).15708

**Published:** 2026-04

**Authors:** Syeda Rawash Mehdi, Anum Liaquat

**Affiliations:** 1 Dr. Syeda Rawash Mehdi, MBBS, Department of Pediatric Surgery, Nishtar Medical University, Multan, Punjab, Pakistan; 2 Dr. Anum Liaquat, MBBS, FCPS, Department of Pediatric Surgery, Nishtar Medical University, Multan, Punjab, Pakistan

**Keywords:** Anastomoses, Intestinal atresia, Multiple atresia, Total parenteral nutrition, Type-IV atresia

## Abstract

Intestinal atresia is one of the most common and complex congenital deformity of neonatal life causing intestinal obstruction. It has four sub-types based on the anatomy of the atresia. Among these four types, Type-IV, which involves multiple atresia segments, is the most severe and rare with high mortality. The biggest surgical challenge is to save enough gut length to prevent long-term complications like short gut syndrome. Early diagnosis and surgical intervention with the right procedure choice is key for survival. Additionally, postoperative neonatal care, parenteral nutrition, and early feeds are crucial for a successful outcome. We present a case report of a newborn with Type-IV intestinal atresia who underwent successful surgical management, highlighting the importance of early diagnosis and prompt intervention with the choice of primary anastomosis.

## INTRODUCTION

The reported incidence of intestinal atresia ranges from 1.3 to 3.5 per 10,000 live births.^1^ Among these, mixed jejunoileal atresia is one of the least common kinds of intestinal atresia encountered. Intestinal atresia is classified into four distinct types, and in our case, it was Type-IV which is constituted of multiple atretic segments across the small intestine. Type-IV atresia is linked with short bowel syndrome, central nervous system dysfunction, and severe immunodeficiency in 25% of cases.^2^ Jejunal atresia typically occurs as a single entity; however, the Type-IV variety which includes multiple atresia, occurs in about 6-20% of atresia cases overall^3^,^4^ and can extend from jejunum to ileum and may even involve duodenum or colon.^3^ This is a relatively rare type with high mortality and morbidity rates, carrying the worst survival rate of 29% compared to 81% of Type-I and others. Thus, it provides a significant level of challenge to a pediatric surgeon.

The operative intervention must be focused on preserving the bowel length to the maximum by any possible method, followed by neonatal intensive care unit and parenteral nutrition.^4^-^6^

The case study describes the Type-IV atresia in a premature neonate with multiple atretic segments and its management strategy leading to the patient’s survival. The purpose of this study was to recognize the importance of early detection and intervention without the burden of stomas and trans anastomotic tube placement^4^,^7^ on the patient as well as to emphasize quality postoperative care for survival in such rare cases.

## CASE DESCRIPTION

In August 2023, a one day old premature baby boy, born via cesarean section at 34 weeks’ gestation, APGAR score eight, presented with meconium not passed since birth, bilious vomiting and abdominal distension. On examination, the abdomen was soft but there was upper abdominal distension with absent bowel sounds. Per rectal exam showed an empty rectum. Prenatal ultrasound at 30 weeks gestation showed distended proximal bowels only and no other significant findings precluding to a diagnosis of atresia. There was an adequate liquor report on ultrasound with no history of polyhydramnios. Xray abdomen erect showed triple bubble sign, pathognomic of proximal intestinal/jejunal atresia. A provisional diagnosis of intestinal atresia was made. Emergency exploratory laparotomy was planned.

After necessary steps to resuscitate and optimize the neonate and adequate preoperative preparation, exploration was done through a supraumbilical right transverse incision. The laparotomy revealed Type-IV jejunoileal atresia about 40 cm distal to the Ligament of Treitz. The multiple atretic sausage-like segments comprised of about two feet of segment followed by approximately one foot segment of normal and patent but collapsed ileum. A tapering jejunoplasty was performed followed by three anastomoses to preserve gut length and a Bishop Koop chimney was made at the site of jejunoplasty to help decompress the gut and prevent pressure on the anastomoses made distally. A stoma formation was not favored so that associated complications can be prevented as well as follow-up surgery. The remaining bowel was irrigated with saline to confirm the patency of distal small and large intestine. Total 10 atresia were confirmed, and three anastomoses were made to prevent short bowel syndrome. The bowel was placed back in the abdominal cavity carefully and closure was done.

**Figure:**
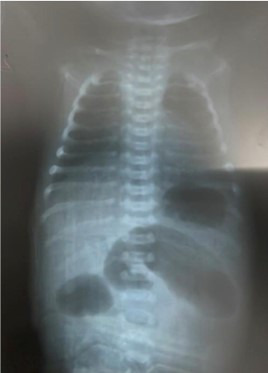
Fig.1:

### Informed consent:

A written statement of informed consent was obtained for writing and publishing of this case study from the patient’s parents.

## RESULTS

Postoperatively, the baby was admitted to the neonatal ICU. Nasogastric tube was placed in situ on aspirate. The baby was provided with vigilant nursing care for a monitored fluid and electrolyte balance, temperature, and signs of sepsis. The chimney began working on 3rd postoperative day with a negligible NG aspirate. Meconium was passed per rectally on 5th postoperative day. He was given total parenteral nutrition for 14 days. In addition, enteral feedings were also started by day five; initially with a simple glucose solution. After confirmation of oral feed tolerance, the patient was switched to Pedialyte after 24 hours and it gradually increased. By day 8, the baby was switched to full strength formula and tolerated well. By day 15, the patient was entirely receiving full strength feedings (milk) and gaining weight and was discharged by day 30. Chimney closure was done on postoperative day 56 on a follow-up.

The patient had follow-ups after two and four weeks initially and then monthly up to the age of one year. By the age of two years, the patient had normal weight and height with overall normal development according to age; and did not exhibit any signs of short bowel syndrome.

**Figure:**
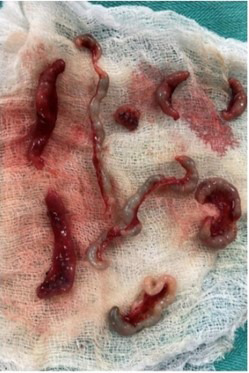
Fig.2:

## DISCUSSION

During our case study, we found multiple atresia and opted for multiple end-to-end anastomosis of the segments to involve most of the atretic gut segments and prevent the short gut complication. We decided against a stoma to reduce the associated complications and patient morbidity. The practice to make a stoma is popular to relieve pressure off the anastomosis and early excretion. But recent data suggests avoiding a stoma yields better patient outcome overall.^1^,^4^,^7^

Intestinal atresia is a complex congenital anomaly. It is one of the most common causes of intestinal obstruction during neonatal life. It is classified into four types based on the location and extent of the atresia. It is thought to result from a vascular accident during fetal development, leading to ischemia and subsequent atresia of the affected bowel segment.^4^,^6^

The Type-IV intestinal atresia, also known as multiple intestinal atresia, is one of the rarest types of atresia. The diagnosis of Type-IV intestinal atresia is challenging. Prenatally, it can be diagnosed with ultrasound, which might show dilated bowel loops, or a fetal MRI.^4^ Postnatally, it can be diagnosed based on the clinical history that includes bilious vomiting, abdominal distension and failure to pass meconium. The investigations available are an abdominal X-ray which shows proximal dilated bowel loops with possible air fluid levels and absence of gas in the rectum. The ultrasound may show dilated fluid filled bowel loops. A contrast enema may help diagnose the level and extent of the atresia.^6^

In Type-IV intestinal atresia, the atretic segments can range anywhere from 9 to 23.^6^ The small intestine is usually affected, but it can sometimes also involve duodenum and large intestines.

Despite recent advances, Type-IV intestinal atresia is still associated with high morbidity and mortality rate. The most common cause of mortality in these patients is sepsis and short bowel syndrome.^6^ The incidence of short bowel syndrome in Type-IV atresia patients reaches up to 65%.^6^ Usually, cases with Type-IV intestinal atresia present during the premature life of a neonate. And care must be taken for the stabilization of such patients both preoperatively and postoperatively.

There are various techniques used for surgical corrections in type IV atresia. They involve chimney formation, stoma formation, resection and anastomosis, multiple anastomosis, tapering enteroplasty, and bowel lengthening procedures.^1^,^4^,^6^,^7^ Stoma formation has usually been the norm in such cases, but it has its own challenges and complications which led us to favor primary anastomosis.^4^ The primary focus of surgical intervention is to save the bowel length to avoid short gut syndrome. All maneuvers must be used to achieve this target. Often the proximal dilated segment is either ischemic or compromised, and sometimes, it may need tapering or resection which might reduce the already short length of the bowel. The most helpful and recommended way to preserve the bowel length is enterotomies and end-to-end anastomosis.^4^,^5^,^7^ Often multiple, the anastomoses involve all the patent and viable atretic segments with the gut.

## CONCLUSION

Type-IV jejunoileal atresia has a high morbidity and mortality index and is often associated with prematurity, short bowel length and sepsis due to enterostasis, hence necessitating urgent work up and prompt surgical intervention to prevent related complications and undue prolonged hospitalization. In the setting of Type-IV atresia, although primary anastomosis is preferred, multiple anastomoses made to prevent short bowel syndrome proved instrumental in reducing the morbidity of the patient. The decision of avoiding stoma and its complications as well as trans anastomotic tubes and associated difficulties proved to be the wiser choice in a complicated case.

As premature patients already have various factors putting them at a disadvantage, they must be protected from all additional and avoidable complications. Our patient outcome highlights the importance of a multidisciplinary approach, including pediatric surgery, anesthesia, and neonatal care.

### Author`s Contribution:

**SRM:** Manuscript writing and responsible for accuracy and integrity of the paper and approval of manuscript

**AL:** Literature search and review and manuscript review.
